# General Medicine and Therapeutics

**Published:** 1896-03

**Authors:** 


### SELECTIONS AND ABSTRACTS.

### GENERAL MEDICINE AND THERAPEUTICS.

EDITED BY DR. LUTHER B. GRANDY.

### MEDICAL EDUCATION, LEGISLATION, ETC.*

*Raising the Standard.*—Medical education in this country is now making one of the longest strides forward that has occurred in its history. This step is the lengthening of the course to three and four years of eight and nine months, instead of the old course of two years of four or five months. We say this step is now being taken, for while many schools have already lengthened their courses during the last eight years, there are others which have arranged to do so soon, and still others which are certain to follow along afterwards.† So the work of higher medical education is still in a progressive state, in that many medical schools have not as yet entered upon their full course of instruction, but it is probable that nearly all of the colleges will have completed this extension during the next four or five years. When they have done so, and one can take a retrospective glance and see that every medical school has added one or two years to its course, and has lengthened the annual session to eight or nine months, the question will naturally arise, What was the moving cause of this extension? Why was it that the medical colleges, which for decades had been contented with a course of two years of four or five months each, began to adopt courses of three or four years of eight or nine months each?

In fact, this question has already been answered by several writers, but the explanations must have come from different standpoints, as they arrive at different conclusions. It is said by some that the Illinois State Board of Health was the prime cause, when it an-

*From advance sheets of the Report of Commissioner of Education. Compiled by A. Erskine Miller.

†The Association of American Colleges, at its meeting in San Francisco, June 7, 1894, "*Resolved*, That students graduating in 1899 or subsequent classes be required to pursue the study of medicine four years and to have attended four annual courses of lectures of not less than six months' duration each."

nounced that no school having a course of less than three years would be considered in good standing and that its graduates could not practice in Illinois without first undergoing an examination. Others say that the medical practice acts adopted in several States during the last five or six years began the work; others attribute it to the resolutions of the American Medical College Association in favor of higher education; others claim that "it was inaugurated by the American Institute of Homeopathy in 1888, when it was ordered that 'After the college sessions of 1890–91 each and all of the homeopathic schools of America will require of their candidates for graduation at least three years of medical study, including three full courses of didactic and clinical instruction of at least six months each.'"

It is probable that many of the leading medical institutions had for some time been in favor of extending the course, but were deterred from fear that their students would leave for competing colleges where less time was required. But when some of the institutions which constituted departments in heavily endowed universities became less dependent upon tuition fees, they were able to adopt longer courses without considering its effect upon attendance. Besides, about this time the number of medical students was becoming unusually large, so that some schools did not really desire an attendance larger than they could well accommodate. By adopting a longer course they would still have as many students as desired, and they would send out graduates better prepared to gain credit for themselves and for the institution.

Many of the State Boards of Health now announce that they will not recognize any institution unless it exacts a full three years' course of study and complies with all other standard requirements of education.* One of the results likely to follow from this elevation of the course is that there will be fewer new colleges coming into existence, for unless an institution has a good standing with the State Boards of Health or examining bodies, it cannot reasonably expect to meet with much success. But, as the present number of medical colleges will be ample for many years to come, no

*Since the above was written the regulations have been made still more stringent. In Oregon, Montana, and Minnesota after 1898 an attendance of four courses of lectures will be required.

complaint will be made because others cannot come into existence. In fact, medical education would be advanced if some institutions already existing were consolidated with others in the same locality.

"The chief difficulty in making a high standard universal lies in the number of the medical colleges. It is, indeed, a sorry admission that the medical schools in this country are the greatest enemy to medical progress, not in themselves, but in their number. . . . The remedy lies in their amalgamation. Let the absurdity cease of small towns having three, four, or six of these struggling institutions, no one of which can have a vigorous life.

"There is now all over the country a growing disposition for the universities to take charge of medical teaching and to develop their medical departments with all the zeal they give to the others. We see it in Michigan; we see it in California and Colorado; we see it in Louisiana and Texas as strikingly as in our Eastern States. There is nothing but good in this. The power, the means, the spirit of the university go out to its branch; the university in turn gains by the reputation of its medical faculty, and by the recognition that medicine is an essential part of the new learning which leads on to the highest attainable civilization."*

*Effect on Attendance of Lengthening the Course.*—The question may be asked, How has the attendance of students been affected by the requirement of another year of study? Adding one year to the course means not only increasing the amount of time required, but also an increase in the financial outlay. It would therefore naturally be expected that many young men would be led to seek other lines of employment. In order to determine this question somewhat definitely the statistics for the last five years have been collected of medical, dental, pharmaceutical, and law students, as all of these have had lengthened courses. Instead of any decrease, however, we find the number of students to have grown steadily larger. In 1888–89 the number of medical students was 15,029; since that time the number has gone steadily forward to 19,752 in 1892–93. As yet even the number of graduates has suffered no special loss, except in the dental schools, where the number of

*Dr. J. M. Da Costa, of Philadelphia.

graduates in 1891–92 was 1,282, and fell to 507 in 1892–93, when the graduating class first encountered the three-year regulation.

Although the actual number of students in attendance during the last five years has regularly increased, it is still very probable that many have been kept out by the lengthened course.

*Legal Control of the Practice of Medicine.*—Unless it be the American Medical College Association, probably nothing has contributed so much to the advancement of medical education in the United States as the establishment of State licensing boards, frequently called boards of health on account of a union of their functions. Here we see how far-reaching and important a little clause sometimes becomes, in a law which directs the licensing boards to register all graduates "of colleges in good standing." Probably no one of the legislators who first voted for the bill containing these words, now found in the law of several States, had any idea of what a powerful leverage they would become in the hands of State licensing boards for stimulating medical colleges to lengthen their course of study and raise the requirements for entrance and graduation. But when the Illinois State Board announced that no medical school would be regarded in good standing which did not require three courses of lectures, the institutions immediately began to fall into line in lengthening their courses, for in truth many schools had favored such an extension before, but were deterred from taking the step because their competitors did not adopt it. Now, however, they recognized the fact that all schools would soon be compelled to advance their standards. This valuable work of State licensing boards was soon recognized in other States, and now we find such boards in thirty-five States and Territories, all of them contributing to a large extent in placing medical education upon a plane where it will reflect honor upon its followers.

It was not many years ago when, in almost any State in the Union, any one could hang out his sign as a medical practitioner and charge as high fees as the best qualified physician in the town. Why, then, should one spend several years in preparing for his work and deplete his purse with the hope of being able to refill it again? It might be said that the meritorious physician would be able eventually to show beyond doubt his qualifications, while the half-educated doctor would be unable to hide his mistakes. That is true to some extent, but the latter generally is able to recognize this fact himself, and when the truth begins to reveal itself to the people, he quietly moves off, possibly to another section of the same city, and begins to plod his old course in a country new. The meritorious doctor then sees that what he expected has taken place, but as a vacancy has occurred, another one soon moves in who has possibly bought out the good will of the departing brother, and then perhaps begins again the identical course of events. Besides, medical knowledge and skill do not alone determine a physician's success; there are other things that help to decide it. An air of self-confidence, a good personal appearance, a cheerful and sociable disposition, and a happy faculty for making acquaintances go far toward securing a reputation as an able physician, and if united with a good degree of dependence on *vis medicatrix naturæ,* he may enjoy many happy days of visiting patients, drawing a veil over ill-timed prescriptions, collecting good fees, and receiving high encomiums from warm-hearted friends.

It may be said that the well qualified physician should also endeavor to possess these external requisites, that they are really as essential in restoring health to the invalid as a well selected prescription; but that is no reason why they should constitute the stock in trade; no reason why the confiding patient should be left to chance or to nature when by the administration of the proper medicine, convalescence would at once begin. But, above all, that is no reason why, so far as can be avoided and when perhaps a life is in danger, dependence should be had upon some one supposed to possess both knowledge and skill, when in fact he has neither. This only illustrates the ease with which deception can be practiced in the healing art, and why the governments of Europe and so many States of this country have adopted regulations to secure at least presumptive evidence that medical practitioners are qualified for their responsible duties. The people have not sufficient time, as individuals, to examine into the qualifications of physicians, to say nothing of the opportunities and knowledge necessary to properly determine such a question. Frequently it is not until some member of the family has been suddenly taken sick that the question arises what physician shall be called, and the query is quite often determined by hurrying to the first drugstore and inquiring of the clerk for the nearest physician.

There are eighteen States in which the diploma of no medical college confers the right to practice, but all candidates for this privilege must undergo an examination. These States are Alabama, Arkansas, Florida, Georgia, Maryland, Minnesota, Mississippi, New Jersey, New York, North Carolina, North Dakota, Pennsylvania, South Carolina, South Dakota, Texas, Utah, Virginia, Washington. In seventeen other States the diploma confers this right only when the board of health or other determining body shall have declared the college to be "in good standing," and this has been interpreted to mean that they are satisfied with the entrance requirements, the length of the term, the number of courses required before graduation, the character of the instruction given, and all other items which aid in fully qualifying the graduate to practice. In some instances State boards have announced in advance that they would recognize no college which did not require so many courses of lectures, or which did not have an annual session of so many months. Medical institutions would then see that they must comply with these requirements or else their graduates from such States would be on unequal footing with those from other schools, and consequently the number of their students would be diminished. In self-defense, therefore, they must comply with all reasonable stipulations.

"A conspicuous effect of these laws has been seen in the improvement of the standard of medical education. To them, more than to any one cause, is due the difference which exists between the condition now and in 1870. In Alabama, Colorado, Connecticut, Illinois, Nebraska, Oregon, South Dakota, and Washington, at least three full courses of five to six months each, no two in the same year, are demanded. The State of Oregon, after 1898, will require four courses of six months each from physicians who wish to practice in that State. There is not only a prolongation of the period of study as the effect of these laws, but there is also an increased demand for a preliminary education, the establishment of new professorships, and more exacting examinations for the degree." (R. H. Fitz, M.D., Boston.)

*Legal Requirements for the Practice of Medicine in the United States.*

| States and Territories. | Requirements. |
|---|---|
| Alabama | Examination by State or county board of medical examiners. |
| Arizona Territory | Registration of diploma. |
| Arkansas | Examination by State or county board of medical examiners. |
| California | Diploma of college " in good standing."* |
| Colorado | Do. |
| Connecticut | Diploma of college " recognized as reputable by one of the chartered medical societies of the State." |
| Delaware | Diploma of " a respectable medical college." |
| District of Colnmbia | Diploma, but practically no requirements. |
| Florida | Examination by State or district board of medical examiners. |
| Georgia | Examination by State board after showing diploma of a college requiring three years of six months. |
| Idaho | Diploma. |
| Illinois | Diploma of college " in good standing." |
| Indiana | Diploma. |
| Iowa | Diploma of college " in good standing." |
| Kansas | Diploma. |
| Kentucky | Diploma of " a reputable college." |
| Louisiana | Diploma of a " medical institution of credit and respectability." |
| Maine | Law recently passed; requirements not known. |
| Maryland | Examination by State board of examiners. |
| Massachusetts | Diploma. |
| Michigan | Do. |
| Minnesota | Examination by State board of examiners. |
| Mississippi | Examination by State board of health. |
| Missouri | Diploma of college " in good standing." |
| Montana | Do. |
| Nebraska | Do. |
| Nevada | Diploma. |
| New Hampshire | No legal requirement. |
| New Jersey | Examination by State board of examiners. |
| New Mexico Territory | Diploma of college " in good standing." |
| New York | Examination by State board of examiners. |
| North Carolina | Do. |
| North Dakota | Do. |
| Ohio | Diploma. |
| Oklahoma | Diploma of college " in good standing." |
| Oregon | Do. |
| Pennsylvania | Examination by State board of examiners. |
| Rhode Island | Diploma " of a reputable and legally chartered medical college, indorsed as such by the State board of health. |
| South Carolina | Examination by State board after presentation of diploma. |
| South Dakota | Examination by State board of health. |
| Tennessee | Diploma of college " in good standing." |
| Texas | Examination by a district board of medical examiners. |
| Utah Territory | Examination of Territorial board. |
| Vermont | Diploma. |
| Virginia | Examination by State board of examiners. |
| Washington | Do. |
| West Virginia | Diploma of " a reputable college." |
| Wisconsin | Diploma. |
| Wyoming | Do. |

In twelve States and Territories it is required only that the candidates for licensure shall present a diploma from some medical school; no question being asked as to the character of the instruc-

*The words " in good standing," as interpreted by the State boards of health and boards of examiners, refer only to those colleges whose regulations comply with the conditions established by these boards.

tion given in the school. This opens a wide door to dishonest evasions of the law, and the traffic in fraudulent diplomas flourishes in consequence. The registrar of medical practitioners frequently has practically no information as to the medical schools of the country, and makes no pretense to examination of the diplomas. But another serious defect in many States is that little or no effort is made to see that unregistered physicians do not practice. The law is left to enforce itself.

An act to regulate the practice of medicine and surgery was lately passed by the legislature of Maine, but its requirements have not yet been ascertained. This leaves New Hampshire as the only State in the Union with no medical-practice law, which it will soon have to enact in self-defense.

In several other States the laws on this subject are of very recent date, showing that the trend of legislation is strongly toward safeguards against incompetent practitioners. In Pennsylvania an act was passed May 18, 1893, to take effect March 4, 1894, requiring an examination before a State board of examiners. In Connecticut the law regulating the practice of medicine only went into effect October 1, 1893, and in Nebraska it was enacted in July, 1891. In South Carolina the law requiring an examination was repealed December 24, 1890, but was re-enacted in December, 1893. The laws of Georgia, Rhode Island, and Maine are of still more recent enactment.

*Study under a Preceptor.*—A few years ago, when nearly all medical colleges had a course of only two years, of four or five months, it was generally stated in the catalogues that the student would be required to spend one year under a preceptor before he could matriculate; and this would have been of much value to him if it was spent under proper direction and attention; but unfortunately those physicians who were most competent to direct his work were generally so engrossed with other duties that they had little time to devote to the medical student. The latter soon recognized the fact that his time was not being well spent, and that he must look after his own interests; consequently he usually spent only a few months, or perhaps weeks, in the study of anatomy, aided only by a few bones which had been hidden away in his preceptor's office. He then went to some medical college where he felt confident he could complete the course in a brief period, registered his name, age, and residence, and the name of his preceptor, paid his matriculation fee, and began to attend the same lectures as those who were soon to complete the course. He soon recognized, however, that he labored under difficulties, for he was nonplussed at the terms spondylolisthesis, symphyseotomy, colporraphy, oöphorectomy, sponge tents, etc., but when he spoke of it to his fellow-students they allayed all fears by telling him he would hear exactly the same thing the next year and would then understand it.

Now that the medical schools have adopted graded courses of three and four years, and the student is led along in his work in an orderly and systematic manner, he is no longer required to spend any time under a preceptor, for it is well known that his time could be far better employed at the institution; in fact the time in a preceptor's office is wasted. "According to Dr. N. S. Davis it (reading medicine in a doctor's office) consisted, in 1877, in little more than the registry of the student's name in the doctor's office, permission to read the books of his library, or not, as he chose, and the giving of a certificate of time of study for the student to take to the medical college when he expected to graduate."

---

### DRY DRESSING FOR THE NAVEL CORD.

Dr. E. A. Tucker says he has seen several cases illustrative of the remarkable fact that pathogenic bacteria entering by the umbilicus may pass over certain viscera and ultimately find a lodgment in some remote part, as in the meninges. The only effective treatment for these infections in the new-born is a prophylactic one, and this consists in carefully dressing the umbilicus with a dry, clean powder. Dr. J. H. Fruitnight said that in the earlier years of his practice, when moist dressings for the cord had been in vogue, he had seen suppuration of the umbilicus much more frequently than since he had adopted the practice of using dry dressings. Several other speakers expressed similar views regarding the efficacy of this treatment of the navel.—*Pediatrics, January* 1, 1896.

